# Automated Platelet Classification Under Different Solvent Conditions Through Deep Learning

**DOI:** 10.3390/jimaging12100519

**Published:** 2026-10-19

**Authors:** Victor O. Daniel, Charlie A. Coupland, Nick Sander, Julius Mboli, Simon D. J. Calaminus, Eva Sousa

**Affiliations:** 1 Centre of Excellence for Data Science, Artificial Intelligence and Modelling, University of Hull, Hull HU6 7RX, UK; victordaniel24@yahoo.com (V.O.D.);; 2 Centre for Biomedicine, Hull York Medical School, University of Hull, Hull HU6 7RX, UKsimon.calaminus@hyms.ac.uk (S.D.J.C.); 3 Tut-All Software GmbH, 76275 Ettlingen, Germany

**Keywords:** deep learning, Convolution Neural Networks, image classification, platelets, zinc, milrinone, Xception

## Abstract

Platelets undergo significant morphological transformations during activation influenced by chemical compounds such as zinc and milrinone. Manual classification methods are subjective, time-consuming, and prone to error. This research aimed to apply and evaluate the efficacy of different pre-trained deep learning Convolution Neural Networks, used for automated binary (treated vs untreated) and multiclass classification (control vs zinc vs milrinone vs combination) of platelet morphological changes induced by these solvents, addressing the limitations of manual classification methods. A dataset of 176 microscopic platelet images from Hull York Medical School was processed, comprising control (n = 43), zinc-treated (n = 45), milrinone-treated (n = 40), and combination treatment (n = 48) samples. Ten pre-trained CNN architectures were evaluated across 166 individual training iterations for both binary and multiclass classification tasks. Progressive optimisation strategies included contrast-limited adaptive histogram equalisation (CLAHE) enhancement, selective layer unfreezing, cross-validation, and hyperparameter tuning. For binary classification, Xception achieved optimal balanced performance (81% accuracy, 76% precision, 74% recall) with proper inter-class discrimination, despite higher numerical metrics (91% accuracy) showing classification bias. For multiclass classification, InceptionV3 and Xception demonstrated superior effectiveness with 72% accuracy. This study demonstrated that Xception and InceptionV3 architectures effectively capture subtle morphological features distinguishing treated platelets, offering reproducible alternatives to subjective manual methods.

## 1. Introduction

Platelets are small anucleate cell fragments circulating in the blood, which are derived from megakaryocytes measuring 2–3 μm in diameter, playing a crucial role in managing vascular integrity, regulating haemostasis and thrombosis [1]. These dynamic cellular elements undergo significant morphological transformations during activation, and they also form clots to arrest bleeding when blood vessels sustain damage [2]. The comprehensive understanding of platelet function and activation mechanisms has far-reaching implications for cardiovascular disease management, stroke prevention, and treatment of bleeding disorders [3]. The functional properties and morphological characteristics of platelets are influenced by various chemical compounds, with zinc (Zn^2+^) playing a particularly significant role in this complex biological system. Approximately 17.3% of the global population experiences inadequate zinc intake, leading to various physiological effects that include bleeding disorders [3]. The significance of zinc in haemostasis is demonstrated by bleeding disorders in zinc-deficient organisms [4] and platelets’ response to Zn^2+^ elevation [5]. Another important compound affecting platelet function is milrinone, a phosphodiesterase-3 inhibitor that increases cyclic adenosine monophosphate (cAMP) levels, thereby inhibiting platelet activation through complex intracellular signalling pathways [6]. The interplay between pro-activating factors like zinc and inhibitory agents like milrinone represents a complex regulatory system that maintains the delicate balance of platelet activity necessary for normal haemostasis while preventing pathological thrombosis. Also, the associated morphological and behavioural changes could signal a defect or pathological condition, making them important for diagnostic purposes [7,8].

Beyond their primary haemostatic function, platelets participate in immune modulation, inflammatory signalling, and wound healing, further broadening the clinical relevance of accurate morphological characterisation [1]. Calaminus et al. identified novel actin-rich structures emerging during the early stages of platelet spreading, illustrating the morphological complexity against which treatment-induced changes must be assessed [9]. Computer-aided systems for blood cell classification have been explored to partially address the limitations of manual analysis [10]; however, their sensitivity to subtle treatment-induced morphological variation in platelets has remained limited without the representational power of deep learning architectures.

The use of traditional methods to identify these changes could be quite subjective, dependent on human expertise, prone to error and time-consuming [2]; however, in recent years, artificial intelligence (AI) and deep learning have transformed medical image analysis, providing sophisticated tools for automated classification of microscopic blood cell images [11]. Within this technological evolution, Convolutional Neural Networks (CNNs) have emerged as particularly effective, demonstrating remarkable performance advantages over traditional machine learning approaches [12]. These advanced deep learning architectures possess the unique capability to extract hierarchical features and learn complex patterns from complex images without human intervention, enabling accurate classification even when confronted with intricate cellular morphologies and subtle structural variations, such as those seen in treated platelets [10,13]. In the application of CNNs for classification, transfer learning has become an indispensable technique in medical image analysis, effectively addressing the persistent challenges of limited dataset availability by leveraging knowledge embedded in pre-trained models [13]. This approach has yielded promising results across diverse medical imaging applications, including the nuanced domain of blood cell classification [11]. The transfer learning approach has proven particularly valuable as it reduces the need for extensive labelled data while potentially improving performance through knowledge transfer from broader image recognition tasks [13,14].

In the application of deep learning models for blood cell classification, several studies have employed various architectures with promising results: Hemalatha et al. achieved 95% accuracy with enhanced CNN models for blood cell classification [12], while Kumar et al. showed U-Net significantly outperformed FCN for platelet segmentation with Dice scores of 0.99 and accuracy of 98% [15]. Notably, Abidoye et al. demonstrated remarkable improvements with data augmentation, where DenseNet121 achieved 81% accuracy on original datasets, while InceptionV3 and DenseNet201 reached 99% and 98% accuracy, respectively, with enhanced augmentation [8]. For other platelet-specific analyses, Kempster et al. developed CNNs that eliminated manual annotation challenges [2], while Poschkamp and Bekeschus achieved 98.4% accuracy in platelet aggregate classification [16]. Chemical influences on platelet morphology have been explored by Coupland et al., who demonstrated zinc’s role as a platelet agonist [3], and Calaminus et al., who identified novel actin-rich structures during platelet spreading [9]. Zhou et al. showed that IPAC (Intelligent Platelet Aggregate Classifier) and CNNs with encoder–decoder architecture can be used to classify platelet aggregates using their distinct morphological features [17]. In distinguishing between COVID-19 and non-COVID-19 thrombosis, a CNN with four convolutional layers was used to phenotypically differentiate non-aggregated platelets, aggregated platelets and WBCs with 96% accuracy [18], and using a hybrid approach of deep learning and image processing techniques via incremental learning, Al-qudah and Suen improved platelet classification accuracy from 82.6% to 98.6% [19]. Ram et al. compared multiple architectures including DenseNet-121, MobileNetV2, and ResNet-50, providing insights into their relative strengths in medical image analysis tasks [20]. Litjens et al. provided a comprehensive survey of deep learning in medical image analysis, documenting strong performance across segmentation, detection, and classification tasks and establishing the methodological foundation upon which platelet-specific applications now build [21]. Ker et al. similarly demonstrated the breadth of deep learning applications in medical imaging, contextualising the expanding scope of automated image analysis in clinical settings [22]. The choice of optimiser is consequential in low-data medical imaging settings: Singh et al. demonstrated that the Adam optimiser provides superior performance for blood cell classification tasks compared to alternatives such as Adadelta and SGD, owing to its adaptive learning rate adjustments and momentum properties [23]. Hutter et al. advocated for progressive hyperparameter refinement rather than exhaustive grid search as best practice for computationally intensive deep learning models, a principle directly applicable to transfer learning workflows in medical imaging [24]. In a comprehensive study, Asghar et al. evaluated the performance of various pre-trained CNN architectures, including VGG16, VGG19, ResNet-50, ResNet-101, ResNet-152, InceptionV3, MobileNetV2, and DenseNet-201 for blood cell classification. Their results demonstrated accuracy rates ranging from 91.375% to 94.72% across different architectures, with ResNet-50 achieving the highest performance. Furthermore, they proposed a custom CNN model that achieved an impressive 99.91% accuracy, highlighting the potential benefits of designing specialised architectures for specific cell classification tasks [10].

This work is supported by the broader context of blood cell classification research. Durant et al. applied very deep CNNs to morphological classification of erythrocytes, demonstrating the generalisability of deep learning across blood cell types [25]. Xu et al. demonstrated a deep CNN for classification of red blood cells in sickle cell anaemia, highlighting the clinical value of automated morphological assessment in haematological disorders [26]. Devi et al. and Ali-Bagido et al. explored white blood cell classification using deep learning, contributing to the growing evidence base for automated haematological image analysis [27,28]. Xia et al. demonstrated automated blood cell detection and counting via deep learning for microfluidic point-of-care medical devices, illustrating the translational potential of these approaches beyond laboratory settings [29]. Naruenatthanaset et al. explored red blood cell segmentation using fuzzy c-means and region growing techniques, further contextualising the methodological progression toward fully automated deep learning solutions in haematological imaging [30].

The role of data augmentation in medical image classification warrants specific consideration in the context of platelet morphology. Shorten and Khoshgoftaar provided a comprehensive survey documenting the general benefits of augmentation for model generalisation [31]. However, Taylor and Nitschke noted that augmentation can introduce artefacts that obscure discriminative features in biological specimens with intricate structural elements [32]. Tellez et al. quantified the effects of augmentation and stain colour normalisation in CNNs for computational pathology, demonstrating that augmentation strategies must be carefully matched to the specific imaging domain [33]. Frid-Adar et al. demonstrated that GAN-based synthetic medical image augmentation provides more effective data enrichment than conventional techniques by learning the underlying data distribution, an approach that may be particularly relevant for specialised microscopic imaging tasks [34]. The challenge of class imbalance further complicates augmentation strategies: Buda et al. conducted a systematic study of the class imbalance problem in CNNs, establishing that standard accuracy metrics are unreliable under imbalance and that confusion matrix analysis is essential for assessing genuine discriminative performance [35]. Japkowicz and Stephen provided a systematic study confirming that models trained on imbalanced datasets tend to minimise loss by defaulting to the majority class, producing misleadingly high accuracy [36]. He and Garcia demonstrated that learning from imbalanced data requires class weighting, specialised loss functions, and careful evaluation metrics [37]. Johnson and Khoshgoftaar further identified that high accuracy can mask significant classification bias in biomedical tasks when evaluation relies solely on aggregate metrics [38].

The architectural properties of specific CNN designs are relevant to their suitability for microscopic platelet image classification. Simonyan and Zisserman established that very deep sequential convolutional networks with 3 × 3 filters achieve strong performance on image classification tasks [39]. He et al. demonstrated that residual skip connections address the vanishing gradient problem, enabling effective training of very deep architectures [40]. Szegedy et al. introduced the Inception architecture employing parallel convolutional paths for multi-scale feature extraction [41]. Huang et al. demonstrated that dense connectivity enabling feature reuse across layers provides strong classification performance [42]. Chollet introduced Xception, leveraging depthwise separable convolutions for efficient feature extraction [43]. Sandler et al. introduced MobileNetV2 with inverted residuals and linear bottlenecks [44]. Tan and Le introduced EfficientNet, demonstrating that compound scaling of network depth, width, and resolution improves performance systematically [45]. Kornblith et al. demonstrated that maintaining input dimensions consistent with pre-training configurations preserves learned feature hierarchies in early layers [46]. Yosinski et al. established that features in deep neural networks transfer across domains with varying effectiveness depending on layer depth, with earlier layers encoding more generalisable and later layers more task-specific representations [47]. Soleimani et al. demonstrated that selective layer unfreezing provides superior model stability compared to fully frozen or completely fine-tuned approaches [48].

Despite advances in deep learning for blood cell classification, significant gaps persist in platelet-specific applications. Research has focused primarily on general blood cell classification and platelet aggregation detection, with limited exploration of treatment-induced morphological changes in platelets. The comparative performance of different CNN architectures for platelet classification remains underexplored, particularly regarding combinations of agents like zinc and milrinone. To address these gaps, this study quantitatively evaluates and compares the efficacy of ten pre-trained CNN architectures for both binary (treated vs untreated) and multiclass (control vs zinc vs milrinone vs milrinone + zinc) classification of microscopic platelet images subjected to zinc, milrinone, and their combination. The research investigates the impact of various data augmentation techniques, regularisation methods, and hyperparameter settings on model performance, with the aim of establishing a reproducible methodology for automated platelet classification that could potentially be applied in clinical and research settings. This research addresses several key questions: how effectively different deep learning architectures can classify platelet morphological changes induced by zinc, milrinone, and their combination; the comparative performances of pre-trained CNN models for binary versus multiclass platelet classification; which CNN architecture provides the optimal balance of accuracy metrics; and how data augmentation and transfer learning techniques affect model performance with limited dataset size. While contributing to AI-assisted medical image analysis and platelet biology, this work acknowledges persistent challenges including dataset imbalance [49], limited ability to detect diverse abnormalities [50], and poor robustness to image variations.

## 2. Materials and Methods

### 2.1. Dataset

The dataset comprised 176 microscopic platelet images from Hull York Medical School, distributed across four experimental conditions: untreated control platelets (n = 43), zinc-treated platelets (n = 45), milrinone-treated platelets (n = 40), and platelets treated with a combination of both agents (n = 48). These treatment conditions were selected to investigate morphological changes induced by compounds known to modulate platelet function through distinct signalling pathways.

**Figure 1:**
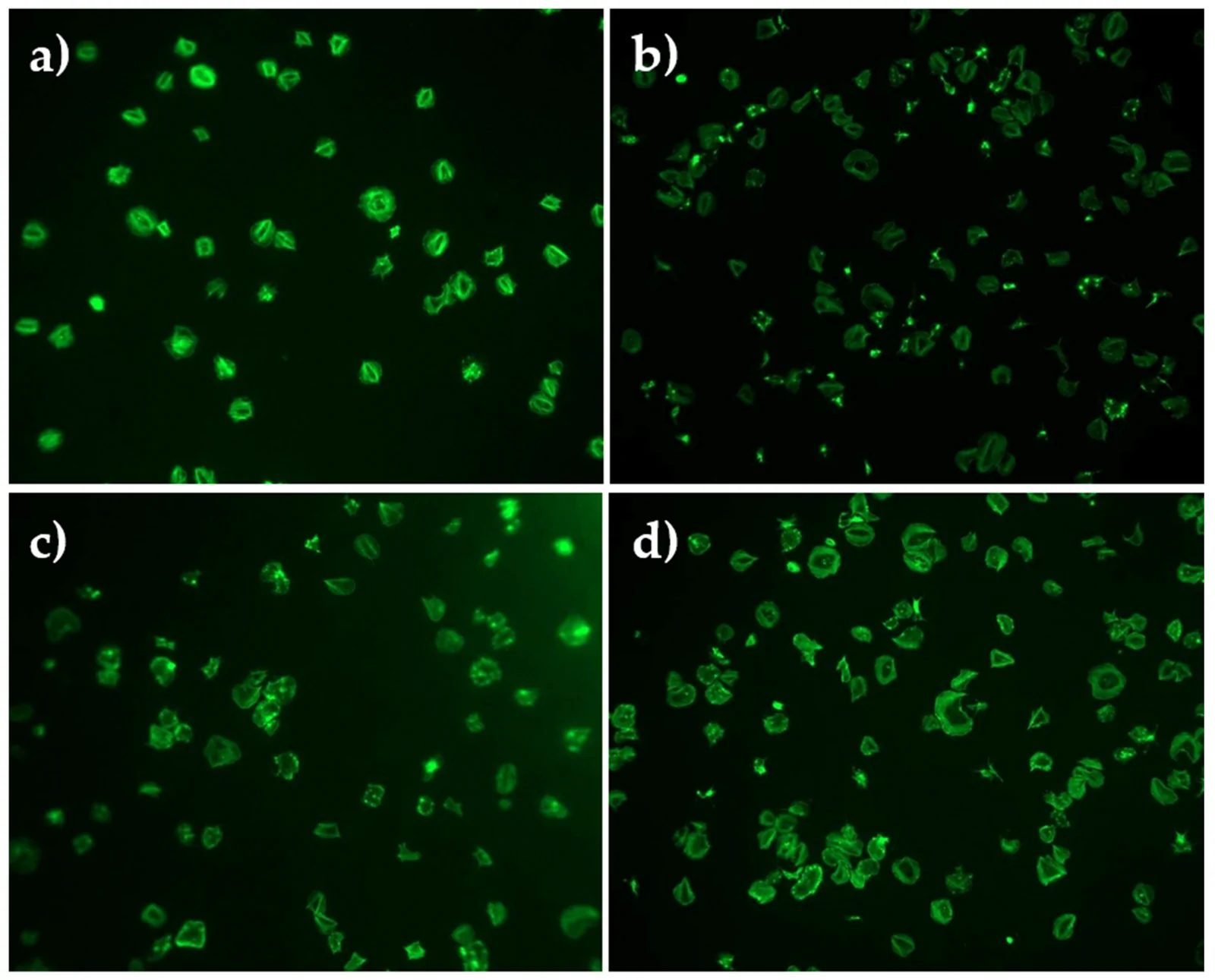
Platelet microscopic images showing (**a**) control (untreated platelets), (**b**) zinc-treated, (**c**) milrinone-treated, and (**d**) zinc- and milrinone-treated.

For the binary classification the images were grouped as ‘Untreated’ (control) versus ‘Treated’ (all treatment conditions) as seen in Figure 1. For the multiclass classification the images maintained the individual treatment categories as seen in Figure 1. Original images in Carl Zeiss Image (CZI) format were converted to Joint Photographic Experts Group (JPEG) and resized from 747 × 600 pixels to 224 × 224 pixels with 3 channels, normalised to a range of [0, 1].

### 2.2. Data Preprocessing

**Figure 2:**
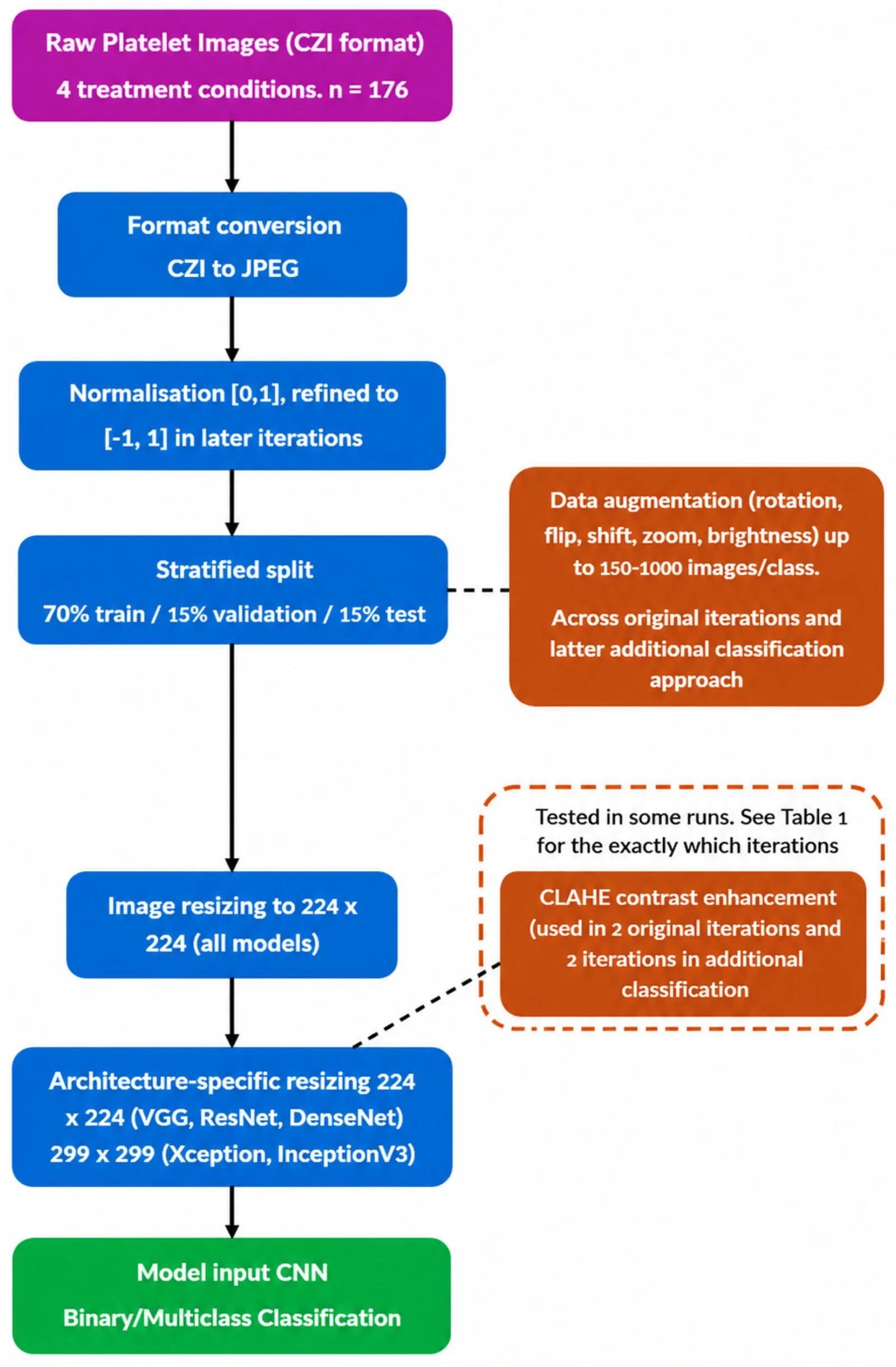
Image preprocessing pipeline applied prior to model training (Table 1).

Prior to model training, each image went through a sequence of preparation steps, illustrated in Figure 2. Raw platelet images originally captured in CZI format were first converted to JPEG for compatibility with standard image loading libraries. Pixel values were normalised, initially to a [0, 1] range and subsequently refined to [−1, 1] to better match the input expectations of the pretrained architecture. The dataset was split into training (70%), validation (15%), and test (15%) sets with stratification to maintain class distribution, given the preexisting class imbalance. Image resizing was applied in two stages across the study. All images were first resized to a fixed 224 × 224 resolution regardless of the target architecture, then resizing was made architecture-specific with 224 × 224for VGG-, ResNet-, and DenseNet-based models, and 299 × 299 for Xception and InceptionV3, to match each network’s native input dimensions. This same preprocessing pipeline was subsequently applied to the additional 2-fold and 3-fold classification approaches described in Section 2.3.3.

**Table 1:** Training criteria of all model training iterations for binary and multiclass classification.

Iteration	Image Loading	Optimiser	Learning Rate	L2 Regularisation (Strength)	Dense Dropout Rate	Batch Size	Epochs	Normalisation	Augmentation (Per Class)	Class Weight	Batch Normalisation	Cross Validation	Unfreeze Layer	Focal Loss	Optimal Threshold	CLAHE
1	224 × 224	Adam	0.0001	No	0.3	32	50	[0, 1]	No	No	No	No	No	No	No	No
2	224 × 224	Adam	0.0001	No	0.3	32	50	[0, 1]	No	Yes	No	No	No	No	No	No
3	224 × 224	Adam	0.0001	No	0.3	32	50	[0, 1]	150	Yes	No	No	No	No	No	No
4	224 × 224	Adam	0.0001	No	0.3	32	50	[0, 1]	200	Yes	No	No	No	No	No	No
5	224 × 224	Adam	0.0001	No	0.3	32	50	[0, 1]	500	Yes	No	No	No	No	No	No
6	224 × 224	Adam	0.0001	No	0.3	32	50	[0, 1]	1000	Yes	No	No	No	No	No	No
7	224 × 224	Adam	0.0001	0.001	0.5	32	50	[0, 1]	No	Yes	Yes	No	No	No	No	No
8	224 × 224	Adam	0.0001	0.001	0.5	32	50	[0, 1]	150	Yes	Yes	No	No	No	No	No
9	224 × 224	Adam	0.0001	0.001	0.5	32	50	[0, 1]	200	Yes	Yes	No	No	No	No	No
10	224 × 224	Adam	0.0001	0.001	0.5	32	50	[0, 1]	500	Yes	Yes	No	No	No	No	No
11	224 × 224	Adam	0.0001	0.001	0.5	32	50	[0, 1]	1000	Yes	Yes	No	No	No	No	No
12	224 × 224	Adam	0.0005	0.001	0.5	16	50	[−1, 1]	No	Yes	Yes	5-fold	15%	No	No	No
13	224 × 224	Adam	0.0005	0.001	0.5	16	50	[−1, 1]	150	Yes	Yes	5-fold	15%	No	No	No
14	224 × 224	Adam	0.0005	0.001	0.5	16	50	[−1, 1]	500	Yes	Yes	5-fold	15%	No	No	No
15	224 × 224	Adam	0.0005	0.001	0.5/0.4	8	75	[−1, 1]	No	Yes	Yes	5-fold	Architecture-specific layer freezing *	Yes	Yes	No
16	224 × 224/299 × 299	Adam	0.0001/ 0.0002	0.1	0.6	16	80	[−1, 1]	No	Yes	Yes	5-fold	20%	No	No	No
17	224 × 224/299 × 299	Adam	0.0001/ 0.0002	0.1	0.6	16	80	[−1, 1]	150	Yes	Yes	5-fold	20%	No	No	No
18	224 × 224/299 × 299	Adam	0.0001/ 0.0002	0.1	0.6	16	100	[−1, 1]	No	Yes	Yes	5-fold	30%	No	No	Yes
19	224 × 224/299 × 299	Adam	0.0001/ 0.0002	0.1	0.6	16	100	[−1, 1]	150	Yes	Yes	5-fold	30%	Yes	Yes	Yes
20	224 × 224	Adam	0.0005	0.001	0.5	16	50	[−1, 1]	No/150	Yes	Yes	5-fold	15%	No	No	No
21	224 ×224	Adam	0.0005	0.001	0.5	8	75	[−1, 1]	No/150	Yes	Yes	5-fold	15%	Yes	No	No
22	224 ×224	Adam	0.0005	0.001	0.5	16	50	[−1, 1]	No/150	Yes	Yes	5-fold	15%	No	No	No
23	224 × 224/299 × 299	Adam	0.001	0.001	0.5	8	100	[−1, 1]	No/150	Yes	Yes	5-fold	15%	Yes	No	No
24	224 × 224/299 × 299	Adam	0.0001/ 0.0002	0.1	0.6	16	100	[−1, 1]	None	Yes	Yes	5-fold	30%	Yes	Yes	Yes
25	224 × 224/299 × 299	Adam	0.0001/ 0.0002	0.1	0.6	16	100	[−1, 1]	None	Yes	Yes	5-fold	30%	Yes	No	Yes

* The proportion of layers unfrozen for fine-tuning has been adjusted based on each architecture’s total depth, rather than using a fixed number of layers across all models.

Beyond this sequence, further preprocessing approaches were done as part of the iterative training procedure, as seen in Table 2. Image augmentation was performed at scales of 150, 200, 500, and 1000 images per class, incorporating 30-degree rotation, width/height shifts (factor 0.2), horizontal/vertical flipping, zoom (factor 0.2), and brightness adjustments (range 0.8–1.2) with ‘nearest’ fill mode. This was done to compensate for the limited size of the original dataset and was tested, with and without, across both the original classification iterations and the later additional classification approach. CLAHE-based contrast enhancement was also introduced to improve local image contrast to help distinguish subtle morphological differences.

### 2.3. Model Architecture and Training

Ten pre-trained CNN models were fine-tuned: Inception-v3 [41], DenseNet-201 [42], MobileNet-v2 [44], ResNet-50 [40], ResNet-101 [40], Xception [43], Inception-ResNet-v2 [51], EfficientNet-b0 [45], VGG-16 [39], and VGG-19 [39]. These architectures differ significantly: VGG models employ sequential designs [39]; ResNet variants implement skip connections [40]; Inception models use parallel convolutional paths [41]; DenseNet-201 enables feature reuse, achieving good performance with far fewer parameter, making them suitable for data scarce settings [42,52]; Xception utilises depthwise separable convolutions [43]; MobileNet-v2 utilises inverted residuals [44]; and EfficientNetB0 optimises scaling [45].

#### 2.3.1. Binary Classification Architecture

**Figure 3:**
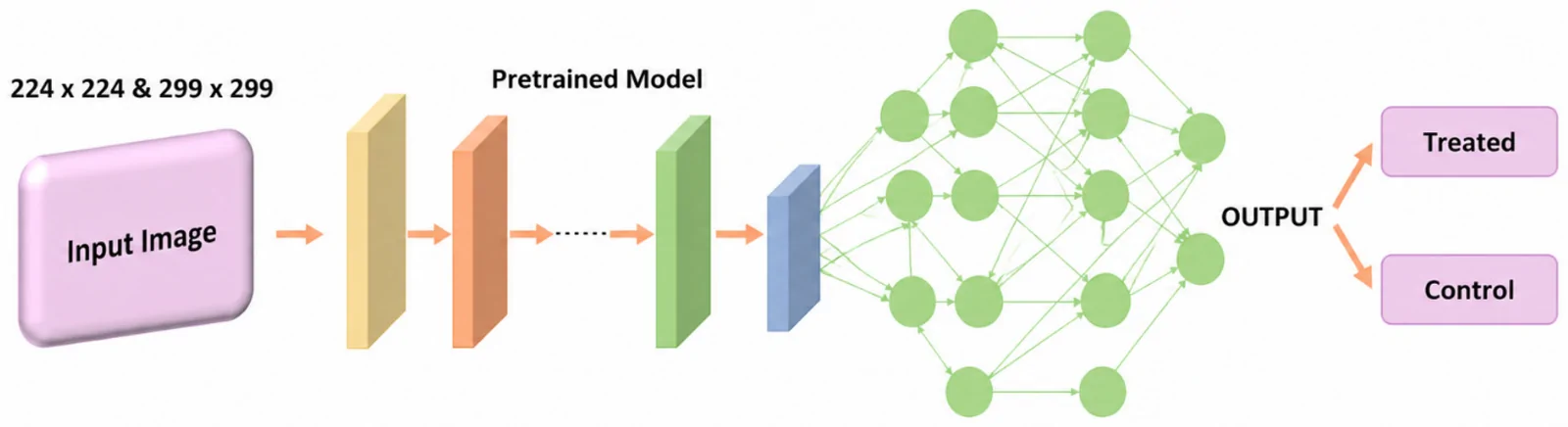
Schematic presentation of the models applied for the binary classification task.

The binary classification was applied to separate platelets in the control group from all the images of the different treatment solvents. For the binary classification task architectures used global average pooling layers followed by dense layers (512 and 256 neurons) with ReLU activation, dropout (0.3), and sigmoid output activation using binary cross-entropy loss. The scheme of the applied methods for binary classification can be seen in Figure 3.

#### 2.3.2. Multiclass Classification Architecture

**Figure 4:**
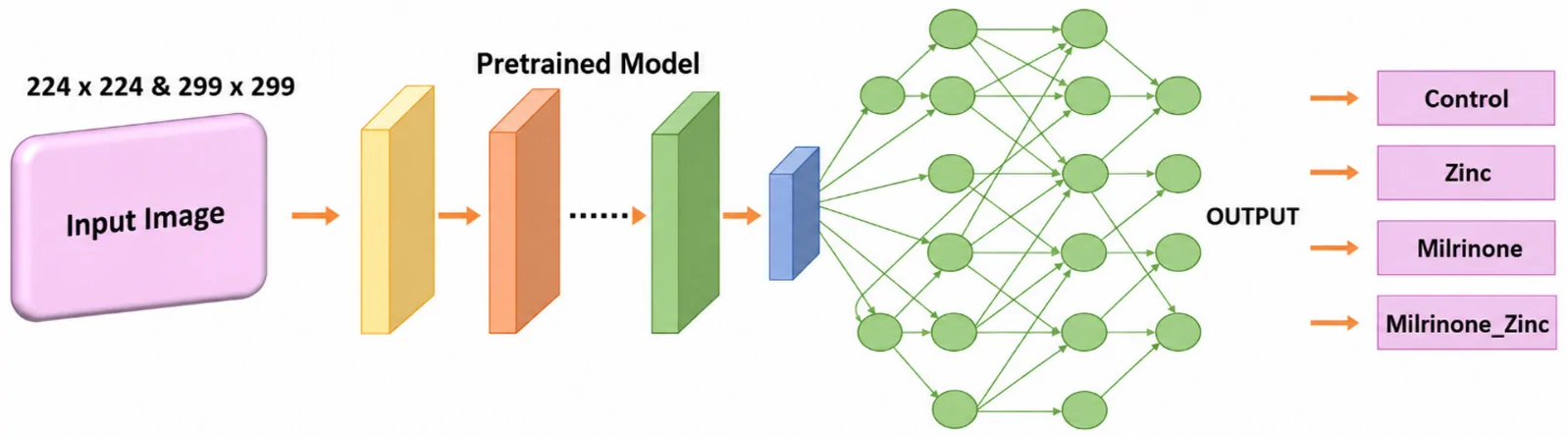
Schematic presentation of the models applied for the multiclass classification task.

The goal in the multiclass classification task was the classification of all the different solvent groups as separate classes. Here, similarly to what was done for the binary classification, the applied architectures had similar structures to the ones used in the binary classification.; however, they used categorical cross-entropy loss and SoftMax activation for a four-class output. The scheme of the applied methods for the multiclass classification can be seen in Figure 4.

#### 2.3.3. Additional Classification Approach

This classification approach was implemented to investigate treatment agent effects on the classification, particularly on the models that struggled with it. This approach aimed to use the training architectures that have performed well for binary and multiclass classification to determine whether morphological changes caused by specific treatment agents were similar and undetectable, thereby resulting in less discrimination by models, or distinct. For this, two approaches were tested. One approach used 2-fold binary classification, using combined control/zinc-treated platelets as one class versus milrinone/milrinone + zinc-treated platelets, and the second one applied a 3-fold multiclass classification with combined control/zinc-treated platelets as one class versus standalone milrinone-treated versus zinc/milrinone combination. In the first of these approaches, the zinc is maintained constant in both classes, so any difference will arise from the difference between control and milrinone; in the second approach, zinc images were combined with control images, and the other groups with milrinone remained independent. This second approach considered the finding of Coupland (2023) where platelets treated with zinc demonstrated great similarity with the control ones [3].

#### 2.3.4. Progressive Optimisation

Models underwent 19 architectural iterations incorporating class weight adjustment, focal loss integration, hyperparameter optimisation, L2 regularisation, batch normalisation, selective layer unfreezing, cross-validation, contrast-limited adaptive histogram equalisation (CLAHE) enhancement [53], custom resolutions (224 × 224/299 × 299), and adaptive thresholding, resulting in 166 individual training configurations for binary classification presented in Table 1.

### 2.4. Evaluation Metrics

Models were evaluated using accuracy, precision, recall, F1-score, and confusion matrices implemented through scikit-learn.

### 2.5. Hardware and Software

Models were trained using Python 3.11 with TensorFlow (v2.18.0) and Keras (v3.8.0) on systems equipped with an Intel^®^ Core i7-8550U CPU, 12GB RAM, and an Nvidia GeForce MX 150 GPU, with additional support from high-performance computers featuring 4× Nvidia Tesla K40m GPUs.

## 3. Results

### 3.1. Binary Classification Results

Initially, the models achieved 74% accuracy, but demonstrated severe classification bias, predicting all samples as the majority ‘Treated’ class, as seen in Table 2. Class weight integration improved performance, with MobileNetV2 achieving 78% accuracy as well as proper classification of both classes.

The introduction of L2 regularisation (0.001), batch normalisation, and increased dropout (0.5) yielded positive results. DenseNet201 and Xception performed best with 78% and 81% accuracy, respectively, as seen in Table 3. Data augmentation unexpectedly degraded performance, likely distorting subtle morphological differences critical for classification.

Architectural refinements incorporating CLAHE enhancement, custom resolutions, 5-fold cross-validation, and 30% deep layer unfreezing achieved the highest metrics. Xception in particular demonstrated very positive performance, as it reached 91% accuracy, 91% precision, and 87% recall, which represented the peak numerical performance achieved. The comparison between these models can be seen in Table 4.

**Figure 5:**
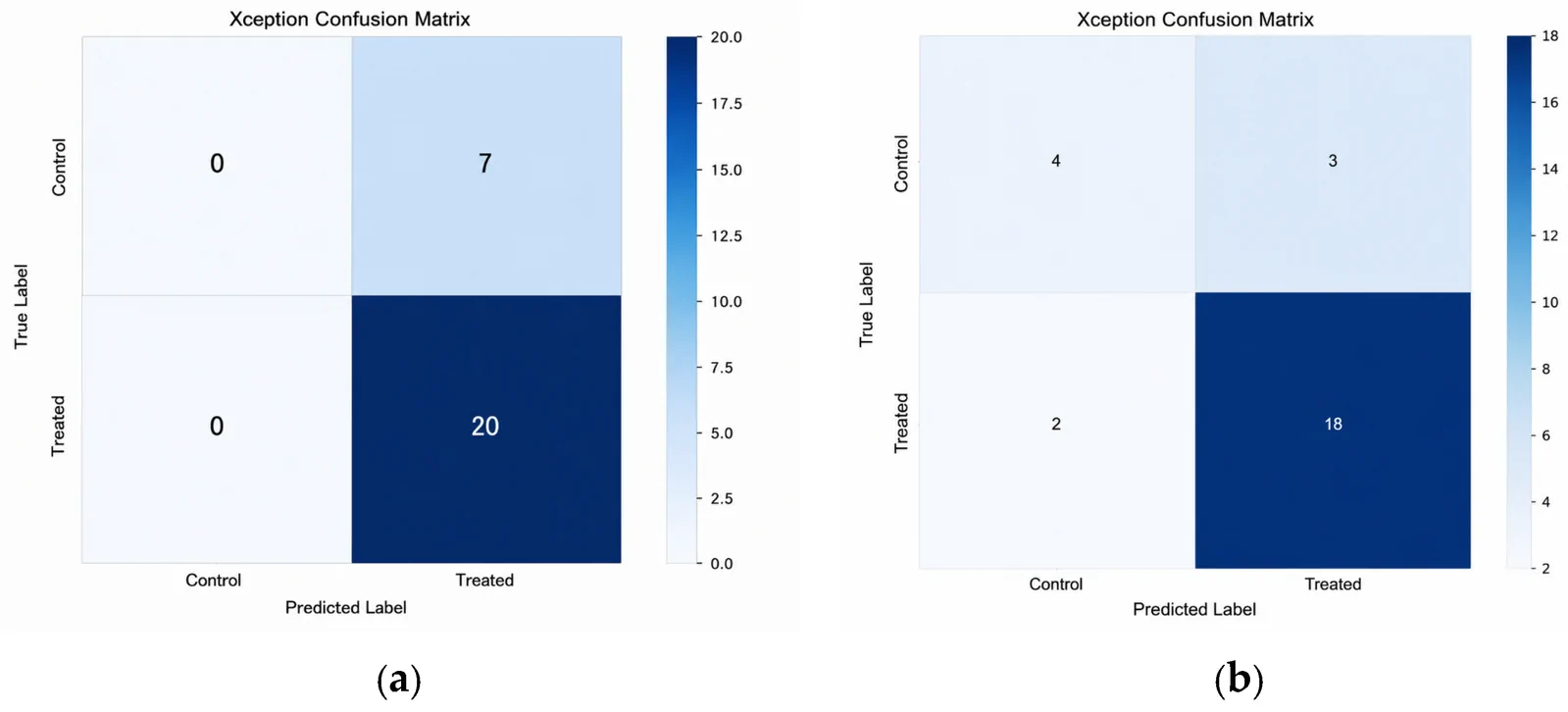
(**a**) Confusion matrix for Xception with single-class prediction bias and (**b**) confusion matrix for Xception with discriminative capacity.

Despite these good results in the measuring metrics, a persistent single-class prediction bias is visible in the confusion matrix presented in Figure 5a. Xception configuration from iteration 7 achieved optimal discrimination with 81% accuracy, 76% precision, and 74% recall while maintaining proper inter-class classification, as seen in Table 3. This represented the best balance between numerical performance and actual discriminative capability, as seen in Figure 5b.

### 3.2. Multiclass Classification Results

Initial models showed poor performance, with Inception-ResNet V2 achieving the highest accuracy at 37%. The addition of class weights and augmentation provided limited improvement. Significant improvements occurred with architectural refinements in iterations 16 and 18, as seen in Table 1. Custom image loading (224 × 224 for DenseNet201/VGG16, 299 × 299 for Xception/InceptionV3) matched each architecture to its native input size, preventing the distortion that occurs when images are resized incorrectly for a given model, 5-fold cross-validation was used to reduce the impact of the small dataset size, giving a more reliable estimate of model performance across different data splits rather than relying on a single train-test division, and selective layer unfreezing (20–30%), which allowed the pretrained models to adjust their higher-level features to the specific characteristics of platelet images, while keeping the early, more general layers unchanged, proved critical. Collectively, these methodological refinements addressed distinct sources of limitations, resulting in marked improvement in performance.

**Figure 6:**
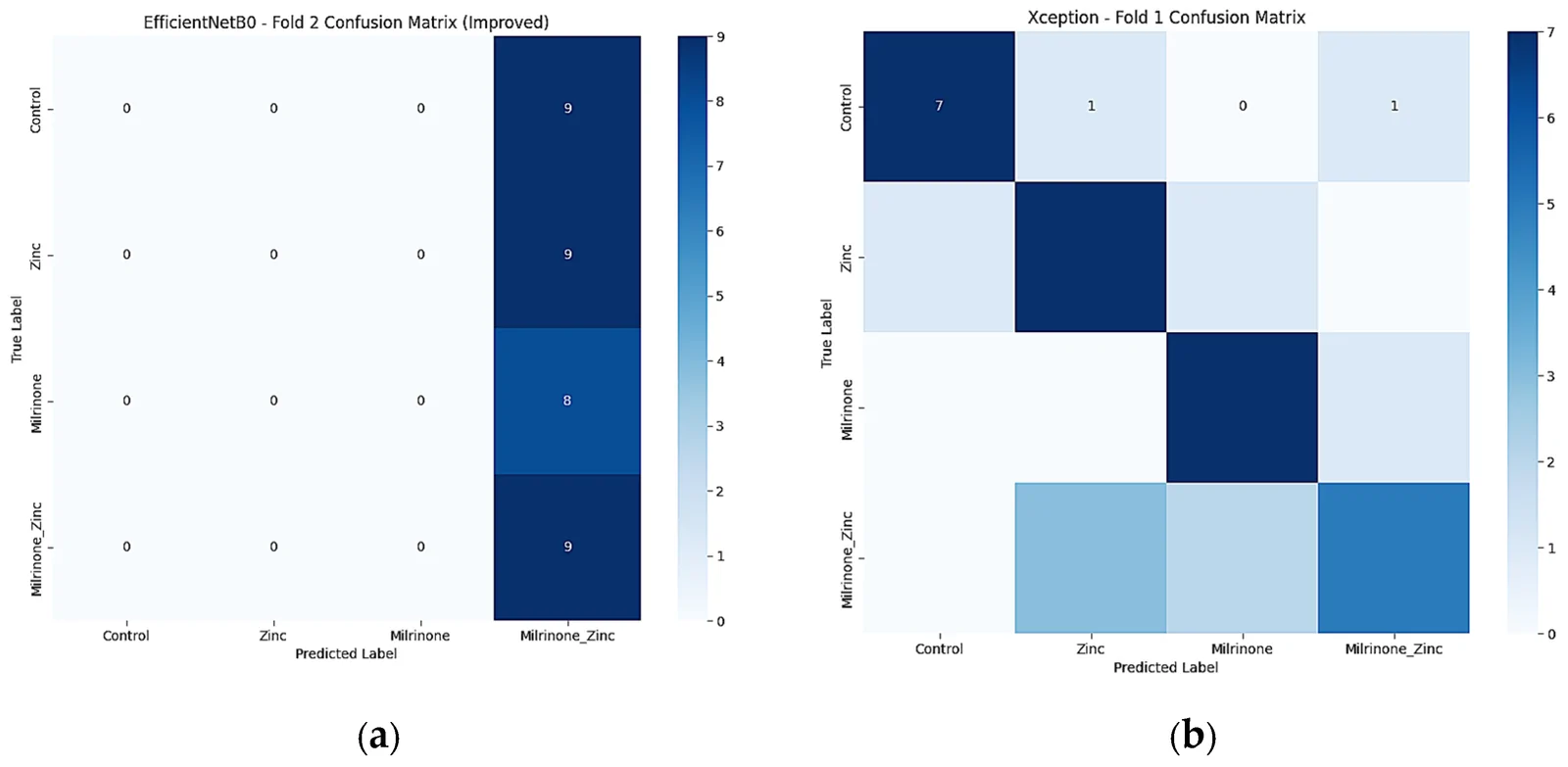
(**a**) Confusion matrix for EfficientNetB0 with single-class prediction bias and (**b**) confusion matrix for Xception with discriminative capacity.

Inception V3 and Xception consistently achieved 70–72% accuracy across multiple optimisation iterations. Iteration 16 delivered the best performance for Xception, with an accuracy of 72%, as seen in Table 5 and Figure 6b.

InceptionV3’s best results were achieved on iteration 18 with CLAHE enhancement, also with an accuracy of 72%, as seen in Table 6. These were the best-performing compositions.

### 3.3. Additional Classification Approaches

On the additional 3-fold classification, combining control/zinc-treated as one class versus milrinone/combination treatments achieved 70% accuracy with Xception, VGG16, InceptionV3, and DenseNet201, suggesting some morphological similarity between control and zinc-treated platelets. Meanwhile, in the 2-fold alternative binary task, the different binary groupings achieved 89% accuracy with InceptionV3, but, similarly to what happened in the original binary classification, the task’s confusion matrices revealed single-class classification bias.

## 4. Discussion

This comprehensive study demonstrates that automated platelet classification using deep learning approaches can effectively distinguish morphological changes induced by chemical treatments, with important implications for both research and clinical applications.

Xception and InceptionV3 architectures consistently demonstrated superior performance across both binary and multiclass tasks. Xception’s depthwise separable convolutions and InceptionV3’s multi-scale feature extraction capabilities appear well-suited for capturing subtle platelet morphological features. The consistent performance of these architectures across different classification scenarios establishes them as optimal choices for platelet classification [10,41,43,51].

Binary classification achieved higher numerical accuracy (up to 91%) but faced significant discrimination challenges due to dataset imbalance. The 81% accuracy with proper discrimination represents a more clinically relevant achievement than the peak accuracy due to imbalanced data.

Multiclass classification, while achieving lower overall accuracy (72%), provided more valuable clinical insights by distinguishing specific treatment effects, highlighting the importance of aligning model development with research objectives. Multiclass classification represented an advancement over binary approaches by distinguishing between specific individual treatments (Control vs zinc vs milrinone vs zinc + milrinone). Iterations 18 and 19 showed promising performance, with InceptionV3 and Xception consistently achieving higher than 70% accuracy.

The difficulty in distinguishing control from zinc-treated platelets was investigated directly through the 3-fold classification approach. Combining control and zinc-treated platelets into a single class achieved over 70% accuracy across multiple architectures, indicating genuine morphological similarities between the two groups. This is consistent with zinc’s known role as a distinct, weaker platelet agonist compared to milrinone’s inhibitory action [4], suggesting that the classifier may be capturing a subtle but real biological difference in activation state and not just a modelling artefact.

Also, the optimum model selection was not only guided by numerical accuracy alone but by discriminative validity as well. The iteration 19 Xception configuration achieved the highest binary classification accuracy; however, its confusion matrix revealed a persistent single-class prediction bias as seen in Figure 5a. In contrast, the iteration 7 Xception configuration achieved lower accuracy but demonstrated good discrimination, correctly distinguishing both control and treated samples rather than defaulting to the majority class as seen in Figure 5b. For an application intended to distinguish genuine biological differences between conditions, correct discrimination between classes offers greater practical value than headline accuracy.

Progressive architectural refinements proved essential for overcoming initial classification bias [24,35]. CLAHE enhancement addressed the specific challenges of microscopic imaging by improving local contrast without amplifying noise [53]. Selective layer unfreezing (30%) enabled adaptation to platelet-specific features while maintaining beneficial pre-trained representations [47,54]. Cross-validation provided robust performance assessment across multiple data splits [55,56]. This targeted optimisation strategy follows best practices outlined by Hutter et al., who advocate for progressive refinement rather than exhaustive grid search when working with computationally intensive deep learning models [24].

Contrary to literature expectations [31,57], traditional data augmentation degraded performance, likely due to distortion of subtle morphological differences critical for platelet classification [58]. This finding aligns with Taylor and Nitschke (2018), who noted that augmentation can obscure discriminative features in specialised medical imaging tasks [32]. The small dataset size (176 images) and class imbalance (43 control vs 133 treated) presented significant challenges requiring careful optimisation strategies [35]. Unlike macroscopic images, where augmentation helps models to learn invariant features, microscopic platelet images contain subtle morphological differences that may be distorted by standard augmentation techniques [31].

Transfer learning combined with selective layer freezing, alongside cross-validation and regularisations, was employed to reduce the risk of overfitting given the dataset size. The additional 3-fold and 2-fold classification approaches provide some further evidence that the models are learning genuine morphological patterns, since regrouping the same images into different class structures produced consistent shifts in performance and discrimination behaviour, as opposed to erratic changes that would be expected from an overfit model latching onto arbitrary features. The risk of overfitting cannot be fully excluded, as the additional classification reused the same images and architectures used earlier in the study. They test class separability more thoroughly, but they are not valid on independent data. All the images came from the same source, and the models have not been tested on images from different microscopy setups, staining protocols, or patient populations. Cross-validation lowers the risk of performance reflecting one favourable split, but it does not replace testing on independently collected data. It is important to note that iteration 7, the preferred binary configuration due to its balanced discrimination, predates the introduction of cross-validation and was evaluated on a single data split.

Vision Transformers (ViT) and other global feature extraction approaches were not evaluated in this study. While pretrained ViT models fine-tuned via transfer learning, similar to the strategy used for the CNN architectures, can partially mitigate their data requirements, ViTs generally still benefit from considerably larger fine-tuning datasets than CNNs to realise their advantage over convolutional inductive biases, given their comparatively weaker locality and translation-invariance priors [59]. Given the modest dataset size used here (176 images across four conditions) and the consistent performance already achieved through CNN-based transfer learning, evaluating transformer-based architectures was considered a lower priority within the scope of this study rather than a methodologically ruled-out option. Comparing pretrained CNN and ViT backbones under matched fine-tuning conditions is a valuable direction for future work.

The ability to automatically distinguish platelet responses to zinc (agonist) and milrinone (inhibitor) treatments has potential applications in cardiovascular research and drug development [3]. Automated platelet classification could streamline assessment of antiplatelet medication effectiveness and identify platelet function disorders more efficiently than manual methods [3,60]. Dataset size and potential unrepresentativeness limit generalisability [35,49,50]. The original 747 × 600 to 224 × 224 pixel resizing may have eliminated critical morphological details that cropping approaches could preserve. Future work should explore custom CNN architectures based on Xception/InceptionV3 designs and ensemble approaches combining multiple top-performing architectures; as explainability grows in importance in the field of healthcare, it would be interesting to include in further studies attention mechanisms for identifying discriminative features.

## 5. Conclusions

This research establishes a comprehensive methodological foundation for automated platelet classification under different solvent conditions using deep learning approaches. Using ten pre-trained CNN architectures across 166 training experiments, it was demonstrated that Xception and InceptionV3 architectures achieve superior performance for both binary and multiclass classification of platelet morphological changes induced by zinc, milrinone, and their combination.

More particularly, it became evident that Xception achieved optimal balanced binary classification (81% accuracy) with proper inter-class discrimination, while Inception V3 and Xception demonstrated consistent multiclass performance (72% accuracy. Preprocessing of the images with CLAHE enhancement has been a crucial step. In addition to that selective layer unfreezing was a critical optimisation strategy, while traditional data augmentation unexpectedly degraded performance in this specialised application.

This automated approach of using CNNs for platelet classification offers reproducible alternatives to subjective manual classification methods, potentially advancing both research applications in platelet biology and clinical diagnostics. While challenges remain regarding dataset limitations and generalisability, this work provides a solid foundation for future development of automated platelet analysis systems that could improve diagnostic efficiency and advance understanding of platelet function in health and disease.

The primary limitation of this study is the modest dataset size of 176 images across four treatment conditions. While transfer learning, 5-fold cross-validation, and regularisation techniques were employed to mitigate overfitting risk, the findings should be interpreted as a methodological proof of concept rather than a clinically validated classifier. Future work should prioritise dataset expansion through multi-site image collection and generative augmentation strategies to improve model robustness and generalisability.

The methodology demonstrated here could be extended to other cellular classification tasks in haematology and adapted for clinical implementation with appropriate validation studies. Future research should focus on expanding datasets, exploring custom architectures, and validating performance across diverse patient populations to fully realise the clinical potential of automated platelet analysis.

## Figures and Tables

**Table 2:** Evaluation metrics for iteration 1 showing all pretrained models for binary classification.

Iteration 1 = Original Architecture				
**Model**	**Accuracy**	**Precision**	**Recall**	**F1 Score**
Binary Classification				
Inception V3	0.7407	0.3704	0.5000	0.4255
DenseNet 201	0.7407	0.3704	0.5000	0.4255
MobileNet V2	0.7407	0.3704	0.5000	0.4255
ResNet 50	0.7407	0.3704	0.5000	0.4255
ResNet 101	0.7407	0.3704	0.5000	0.4255
Xception	0.7407	0.3704	0.5000	0.4255
InceptionResNet V2	0.7407	0.3704	0.5000	0.4255
EfficientNet B0	0.7407	0.3704	0.5000	0.4255
VGG16	0.7407	0.3704	0.5000	0.4255
VGG19	0.7407	0.3704	0.5000	0.4255

**Table 3:** Evaluation metrics for iteration 7 showing all pretrained models for binary classification.

Model	Accuracy	Precision	Recall	F1 Score
Iteration 7				
Binary Classification				
Inception V3	0.7407	0.3704	0.5000	0.4255
DenseNet 201	0.7778	0.8846	0.5714	0.5598
MobileNet V2	0.4444	0.4533	0.4393	0.4156
ResNet 50	0.7407	0.3704	0.5000	0.4255
ResNet 101	0.7407	0.3704	0.5000	0.4255
Xception	0.8148	0.7619	0.7357	0.7467
Inception-ResNet V2	0.7407	0.3704	0.5000	0.4255
EfficientNet B0	0.7407	0.3704	0.5000	0.4255
VGG16	0.7407	0.3704	0.5000	0.4255
VGG19	0.7407	0.3704	0.5000	0.4255

**Table 4:** Evaluation metrics for iteration 19 showing the best-performing models for binary classification.

Model	Accuracy	Precision	Recall	F1 Score
Iteration 19 = Iteration 18 + Focal Loss + Adaptive Threshold (for Binary classification) + Augmentation (with new parameter values)				
Binary Classification				
Inception V3	0.8635 ± 0.0585	0.8946 ± 0.0538	0.7355 ± 0.1223	0.7557 ± 0.1325
DenseNet 201	0.8862 ± 0.0654	0.8859 ± 0.0513	0.8323 ± 0.1425	0.8178 ± 0.1476
Xception	0.9148 ± 0.0256	0.9059 ± 0.0494	0.8732 ± 0.0509	0.8806 ± 0.0350
VGG16	0.8071 ± 0.0568	0.6818 ± 0.2541	0.6185 ± 0.1079	0.6080 ± 0.1588

**Table 5:** Evaluation metrics for iteration 16 showing focused pretrained models for multiclass classification.

Model	Accuracy	Precision	Recall	F1 Score
**Iteration 16 = Custom Model Image Loading + Cross Validation + Unfreeze 20% + Optimal LR**				
Multiclass Classification				
Inception V3	0.6990 ± 0.0907	0.7243 ± 0.0858	0.6978 ± 0.0886	0.6906 ± 0.0920
DenseNet 201	0.5905 ± 0.0687	0.6152 ± 0.0806	0.5926 ± 0.0687	0.5882 ± 0.0642
Xception	0.7216 ± 0.0835	0.7581 ± 0.0796	0.7199 ± 0.0852	0.7130 ± 0.0889
VGG16	0.6249 ± 0.0496	0.6967 ± 0.0439	0.6303 ± 0.0529	0.6177 ± 0.0540

**Table 6:** Evaluation metrics for iteration 18 showing focused pretrained models for multiclass classification.

Model	Accuracy	Precision	Recall	F1 Score
**Iteration 18 = Custom Model Image Loading + CLAHE + Cross Validation + Unfreeze 30% + Optimal LR**				
Multiclass Classification				
Inception V3	0.7216 ± 0.0609	0.7543 ± 0.0590	0.7238 ± 0.0641	0.7187 ± 0.0654
DenseNet 201	0.6703 ± 0.0844	0.6882 ± 0.1020	0.6681 ± 0.0796	0.6616 ± 0.0926
Xception	0.6476 ± 0.0470	0.7167 ± 0.0521	0.6472 ± 0.0479	0.6444 ± 0.0488
VGG16	0.4900 ± 0.1690	0.4166 ± 0.2677	0.4821 ± 0.1659	0.4021 ± 0.2208

## Data Availability

The data presented in this study are available on request from the corresponding author.

## Abbreviations

sAI	Artificial Intelligence
cAMP	Cyclic Adenosine Monophosphate
CLAHE	Contrast-Limited Adaptive Histogram Equalisation
CNN	Convolutional Neural Network
CNNs	Convolutional Neural Networks
CPU	Central Processing Unit
CZI	Carl Zeiss Image
FCN	Fully Convolutional Network
GAN	Generative Adversarial Network
GB	Gigabyte
GPU	Graphics Processing Unit
GPUs	Graphics Processing Units
IPAC	Intelligent Platelet Aggregate Classifier
JPEG	Joint Photographic Experts Group
L2	L2 Regularisation
LR	Learning Rate
RAM	Random Access Memory
ReLU	Rectified Linear Unit
SGD	Stochastic Gradient Descent
WBCs	White Blood Cells

## References

[1] Ghoshal K., Bhattacharyya M. (2014). Overview of platelet physiology: Its haemostatic and non-haemostatic role in disease pathogenesis. Sci. World J..

[2] Kempster C., Butler G., Kuznecova E., Taylor K.A., Kriek N., Little G., Sowa M.A., Sage T., Johnson L.J., Gibbins J.M. (2022). Fully automated platelet differential interference contrast image analysis via deep learning. Sci. Rep..

[3] Coupland C.A., Naylor-Adamson L., Booth Z., Price T.W., Gil H.M., Firth G., Avery M., Ahmed Y., Stasiuk G.J., Calaminus S.D.J. (2023). Platelet zinc status regulates prostaglandin-induced signalling, altering thrombus formation. J. Thromb. Haemost..

[4] Watson B.R., White N.A., Taylor K.A., Howes J.M., Malcor J.D., Bihan D., Sage S.O., Farndale R.W., Pugh N. (2016). Zinc is a transmembrane agonist that induces platelet activation in a tyrosine phosphorylation-dependent manner. Metallomics.

[5] Kuravi S.J., Ahmed N.S., Taylor K.A., Capes E.M., Bye A., Unsworth A.J. (2023). Delineating Zinc Influx Mechanisms during Platelet Activation. Int. J. Mol. Sci..

[6] Francis G.S., Bartos J.A., Adatya S. (2014). Inotropes. J. Am. Coll. Cardiol..

[7] Qiu X., Nair M.G., Jaroszewski L., Godzik A. (2024). Deciphering Abnormal Platelet Subpopulations in COVID-19, Sepsis and Systemic Lupus Erythematosus through Machine Learning and Single-Cell Transcriptomics. Int. J. Mol. Sci..

[8] Abidoye I., Ikeji F., Coupland C.A., Calaminus S.D.J., Sander N., Sousa E. (2025). Platelets Image Classification Through Data Augmentation: A Comparative Study of Traditional Imaging Augmentation and GAN-Based Synthetic Data Generation Techniques Using CNNs. J. Imaging.

[9] Calaminus S.D.J., Thomas S., McCarty O.J.T., Machesky L.M., Watson S.P. (2023). Identification of a novel, actin-rich structure, the actin nodule, in the early stages of platelet spreading. J. Thromb. Haemost..

[10] Asghar R., Kumar S., Hynds P. (2024). Automatic classification of 10 blood cell subtypes using transfer learning via pre-trained convolutional neural networks. Inform. Med. Unlocked.

[11] Asghar R., Kumar S., Hynds P., Mahfooz A. (2023). Classification of All Blood Cell Images using ML and DL Models. J. Appl. Soft Comput..

[12] Hemalatha B., Karthik B., Krishna Reddy C.V., Latha A. (2022). Deep learning approach for segmentation and classification of blood cells using enhanced CNN. Meas. Sens..

[13] Kim H.E., Cosa-Linan A., Santhanam N., Jannesari M., Maros M.E., Ganslandt T. (2022). Transfer learning for medical image classification: A literature review. BMC Med. Imaging.

[14] Pan S.J., Yang Q. (2010). A Survey on Transfer Learning. IEEE Trans. Knowl. Data Eng..

[15] Kumar A., Coupland C.A., Vaz T.F., Jones W., Valcarce-Dineiro R., Calaminus S.D.J., Sousa E. (2024). UNet as a deep learning-based method for platelets segmentation in microscopic images. bioRxiv.

[16] Poschkamp B., Bekeschus S. (2024). Convolutional neuronal network for identifying single-cell-platelet-platelet-aggregates in human whole blood using imaging flow cytometry. Cytometry.

[17] Zhou Y., Yasumoto A., Lei C., Huang C.J., Kobayashi H., Wu Y., Yan S., Sun C.W., Yatomi Y., Goda K. (2020). Intelligent classification of platelet aggregates by agonist type. eLife.

[18] Zhang Z., Chen P., McGough M., Xing F., Wang C., Bui M., Xie Y., Sapkota M., Cui L., Dhillon J. (2017). Pathologist-level interpretable whole-slide cancer diagnosis with deep learning. Nat. Mach. Intell..

[19] Al-qudah M., Suen C.Y. (2021). Enhanced deep learning approach for blood cells platelet classification. Int. J. Pattern Recognit. Artif. Intell..

[20] Ram S., Vinoth S., Gopalakrishnan R.N., Balakumar A.A., Kalinathan L., Velankanni T.A.J. (2024). Leveraging Diverse CNN Architectures for Medical Image Captioning: DenseNet-121, MobileNetV2, and ResNet50 in ImageCLEF 2024. CLEF Work. Notes.

[21] Litjens G., Kooi T., Bejnordi B.E., Setio A.A.A., Ciompi F., Ghafoorian M., Van Der Laak J.A., Van Ginneken B., Sánchez C.I. (2017). A survey on deep learning in medical image analysis. Med. Image Anal..

[22] Ker J., Wang L., Rao J., Lim T. (2017). Deep learning applications in medical image analysis. IEEE Access.

[23] Singh R., Mahajan A., Sachan M., Kumar A. Impact of Adam, Adadelta, SGD on CNN for White Blood Cell Classification. Proceedings of the 2020 IEEE International Symposium on Signal Processing and Information Technology.

[24] Hutter F., Kotthoff L., Vanschoren J. (2019). Automated Machine Learning: Methods, Systems, Challenges.

[25] Durant T.J., Olson E.M., Schulz W.L., Torres R. (2017). Very deep convolutional neural networks for morphologic classification of erythrocytes. Clin. Chem..

[26] Xu M., Papageorgiou D.P., Abidi S.Z., Dao M., Zhao H., Karniadakis G.E. (2017). A deep convolutional neural network for classification of red blood cells in sickle cell anaemia. PLoS Comput. Biol..

[27] Devi S.S., Singh N.H., Devi K.D. A novel approach to white blood cell classification. Proceedings of the International Conference on Computing, Communication and Networking Technologies.

[28] Ali-Bagido R., Alzahrani M., Arif M. (2021). White Blood Cell Types Classification Using Deep Learning Models. IJCSNS Int. J. Comput. Sci. Netw. Secur..

[29] Xia T., Jiang R., Fu Y.Q., Jin N. (2019). Automated Blood Cell Detection and Counting via Deep Learning for Microfluidic Point-of-Care Medical Devices. IOP Conf. Ser. Mater. Sci. Eng..

[30] Naruenatthanaset K., Chalidabhongse T.H., Palasuwan D., Anantrasirichai N. Red blood cells segmentation using fuzzy c-means and region growing. Proceedings of the IEEE 3rd Global Conference on Life Sciences and Technologies.

[31] Shorten C., Khoshgoftaar T.M. (2019). A survey on Image Data Augmentation for Deep Learning. J. Big Data.

[32] Taylor L., Nitschke G. Improving deep learning with generic data augmentation. Proceedings of the 2018 IEEE Symposium Series on Computational Intelligence.

[33] Tellez D., Litjens G., Bándi P., Bulten W., Bokhorst J.M., Ciompi F., Van Der Laak J. (2019). Quantifying the effects of data augmentation and stain colour normalisation in convolutional neural networks for computational pathology. Med. Image Anal..

[34] Frid-Adar M., Diamant I., Klang E., Amitai M., Goldberger J., Greenspan H. (2018). GAN-based synthetic medical image augmentation for increased CNN performance in liver lesion classification. Neurocomputing.

[35] Buda M., Maki A., Mazurowski M.A. (2018). A systematic study of the class imbalance problem in convolutional neural networks. Neural Netw..

[36] Japkowicz N., Stephen S. (2002). The class imbalance problem: A systematic study. Intell. Data Anal..

[37] He H., Garcia E.A. (2009). Learning from imbalanced data. IEEE Trans. Knowl. Data Eng..

[38] Johnson J.M., Khoshgoftaar T.M. (2019). Survey on deep learning with class imbalance. J. Big Data.

[39] Simonyan K., Zisserman A. (2015). Very Deep Convolutional Networks for Large-Scale Image Recognition. Int. Conf. Learn. Represent..

[40] He K., Zhang X., Ren S., Sun J. Deep Residual Learning for Image Recognition. Proceedings of the IEEE Conference on Computer Vision and Pattern Recognition.

[41] Szegedy C., Vanhoucke V., Ioffe S., Shlens J., Wojna Z. Rethinking the Inception Architecture for Computer Vision. Proceedings of the IEEE Conference on Computer Vision and Pattern Recognition.

[42] Huang G., Liu Z., Van Der Maaten L., Weinberger K.Q. Densely Connected Convolutional Networks. Proceedings of the IEEE Conference on Computer Vision and Pattern Recognition.

[43] Chollet F. Xception: Deep Learning with Depthwise Separable Convolutions. Proceedings of the IEEE Conference on Computer Vision and Pattern Recognition.

[44] Sandler M., Howard A., Zhu M., Zhmoginov A., Chen L.C. MobileNetV2: Inverted Residuals and Linear Bottlenecks. Proceedings of the IEEE Conference on Computer Vision and Pattern Recognition.

[45] Tan M., Le Q. (2019). EfficientNet: Rethinking Model Scaling for Convolutional Neural Networks. Int. Conf. Mach. Learn..

[46] Kornblith S., Shlens J., Le Q.V. Do better ImageNet models transfer better?. Proceedings of the IEEE/CVF Conference on Computer Vision and Pattern Recognition 2019.

[47] Yosinski J., Clune J., Bengio Y., Lipson H. (2014). How transferable are features in deep neural networks?. Adv. Neural Inf. Process. Syst..

[48] Soleimani M., Ismail N.A., Talib M.A. (2023). Frozen or Fine-tuned? Analysing Deep Learning Models and Training Strategies for Optimising Big Five Personality Traits Prediction from Text. Comput. Methods Programs Biomed..

[49] Wong A., Anantrasirichai N., Chalidabhongse T.H., Palasuwanc D., Palasuwand A., Bull D. (2021). Analysis of Vision-based Abnormal Red Blood Cell Classification. ResearchGate.

[50] Hegde R.B., Prasad K., Hebbar H., Singh B.M.K. (2018). Development of a robust algorithm for detection of nuclei and classification of white blood cells in peripheral blood smear images. J. Med. Syst..

[51] Szegedy C., Ioffe S., Vanhoucke V., Alemi A. Inception-v4, Inception-ResNet and the Impact of Residual Connections on Learning. Proceedings of the Thirty-First AAAI Conference on Artificial Intelligence.

[52] Khened M., Kollerathu V.A., Krishnamurthi G. (2019). Fully convolutional multi-scale residual DenseNets for cardiac segmentation and automated cardiac diagnosis using ensemble of classifiers. Med. Image Anal..

[53] Pizer S.M., Johnston R.E., Ericksen J.P., Yankaskas B.C., Muller K.E. Contrast-Limited Adaptive Histogram Equalization: Speed and Effectiveness. Proceedings of the First Conference on Visualization in Biomedical Computing.

[54] Guo Y., Shi H., Kumar A., Grauman K., Rosing T., Feris R. (2018). SpotTune: Transfer Learning through Adaptive Fine-tuning. arXiv.

[55] Wilimitis D., Walsh C.G. (2023). Practical Considerations and Applied Examples of Cross-Validation for Model Development and Evaluation in Health Care: Tutorial. JMIR AI.

[56] Kohavi R. A Study of Cross-Validation and Bootstrap for Accuracy Estimation and Model Selection. Proceedings of the 14th International Joint Conference on Artificial Intelligence.

[57] Perez L., Wang J. (2017). The Effectiveness of Data Augmentation in Image Classification using Deep Learning. arXiv.

[58] Yang S., Xiao W., Zhang M., Guo S., Zhao J., Shen F. (2023). Image Data Augmentation for Deep Learning: A Survey. arXiv.

[59] Dosovitskiy A., Beyer L., Kolesnikov A., Weissenborn D., Zhai X., Unterthiner T., Dehghani M., Minderer M., Heigold G., Gelly S. An Image is Worth 16x16 Words: Transformers for Image Recognition at Scale. Proceedings of the International Conference on Learning Representations.

[60] Yoon I., Han J.H., Jeon H.J. (2024). Advances in Platelet-Dysfunction Diagnostic Technologies. Biomolecules.

